# Changes in income-related inequalities in cervical cancer screening during the Spanish economic crisis: a decomposition analysis

**DOI:** 10.1186/s12939-018-0894-x

**Published:** 2018-12-13

**Authors:** María Merino-Ventosa, Rosa M. Urbanos-Garrido

**Affiliations:** 1 Department of Health Outcomes Research, Weber, Economía y Salud, Madrid, Spain; 2 Department of Applied Economics, Public Economics and Political Economy, School of Economics, Complutense University of Madrid, Campus de Somosaguas, 28223 Pozuelo de Alarcón, Madrid, Spain

**Keywords:** Cervical cancer screening, Income-related inequality, Spain, Economic crisis, H51, I14, I18

## Abstract

**Background:**

Cervical cancer is one of the most prevalent cancers, but it may be prevented by early detection. Social inequalities in the use of cytology testing have been identified in the literature. However, the degree of income-related inequality has not been quantified and determinants of inequality changes during the economic crisis remain unknown.

**Methods:**

Using the Spanish National Health Surveys (2006–07 / 2011–12), we analyzed how income-related inequalities in the use of cervical cancer screening for women aged 25–64 changed across the economic crisis. We used corrected concentration indices (CCI) which were further decomposed in order to compute the contribution of the explanatory variables. An Oaxaca-type approach was employed to investigate the origin of changes over time.

**Results:**

Our final sample consisted of 10,743 observations in 2006–07 and 6587 in 2011–12. Despite the higher prevalence of screening over time (from 73.9 to 77.9%), pro-rich inequality significantly increased (from CCI = 0.1726 to CCI = 0.1880, *p* < 0.001). Income was the main determinant of inequality in cervical screening, although its contribution decreased over time, as well as the contribution of the type of health insurance, mainly due to changes in elasticity. Other factors, such as nationality or the educational level, seem to have played an important role in the increase of pro-rich inequality of cytology testing.

**Conclusions:**

Reducing cervical screening inequalities would require actions focused on most vulnerable groups such as migrants, low income and low educated population. The implementation of population-based screening programs would also help to cope with income-related inequalities in cytology testing.

## Background

Cervical cancer is the fourth most common cancer in women worldwide (569,847 new cases, 6.6% out of total in 2018) and the eighth most common cancer overall (3.2% out of total), according to data from the World Health Organization [1]. In 2018, there were 311,365 estimated deaths from cervical cancer worldwide, accounting for 7.5% of all cancer deaths in females [1].

In Spain, 2584 new cases of cervical cancer were estimated for 2017, which represents 2.8% of all new female cancer cases, and the number of deaths reached 620 in 2016 (1.4% of all female cancer deaths) [2]. Nowadays, Spain is one of the countries with the lowest incidence rates in the European Union, and also with one of the lowest rates of mortality [3]. The reduction of incidence in recent years has been generally observed in high-income countries [4].

Between one third and one half of cancer deaths can be avoided with prevention, early detection and treatment [5]. The European guidelines for quality assurance in cervical cancer screening agree with implementing national population-based screening programs [6]. At a regional level, some studies have shown that a cervical smear performed regularly is effective to improve the secondary prevention [7, 8], while other studies have not found it cost-effective or suggested to redefine inclusion criteria [9, 10]. In the Spanish National Health Service, where health care is regionally managed and delivered, prevention strategies developed in each region may differ. Screening programs are, unlike in other European countries, such as Italy, Netherlands or Sweden, mostly opportunistic (i.e. not systematically offered to target population, which usually lead to over-screening in more motivated women, and also to under-screening in less informed women) [3], and their characteristics and inclusion criteria vary (for instance, some regional health services recommend to undertake a cytology every three years for all women aged 25–65, whilst others widen that period to five years for those aged over 35). Although both types of screening can reduce the incidence of cervical cancer, population-based ones show more equity and effectiveness [11–15]. At the present time, only three Spanish regions have implemented a population-based screening program (Castilla y León, La Rioja and, more recently, Castilla-La Mancha) [16–18], although some other regional governments have announced that they will also do during next years [19–21].

The relationship between the use of health preventive services and different socio-demographic variables has been widely addressed in the literature. Previous studies have highlighted the relevance of socioeconomic variables on the probability of making use of breast and cervical screening [14, 15, 22–29], and some have identified those factors contributing to income-related inequality of cervical screening [22]. More recently, the evolution of frequency of cervical cytology testing, as well as its determinants, has also been studied [30]. However, the degree of income-related inequality has not been quantified and determinants of inequality changes during the economic crisis remain unknown.

Spain has been one of the European countries where the Great Recession had a more remarkable impact on the health care system. Particularly, Spanish austerity policies lead to a decrease around 13% in public health expenditure from 2009 to 2013, and implied the implementation of several reforms, including a change in the existing entitlement rules [31]. Spending cuts translated into an increase of waiting times and waiting lists. Waiting times for first visits to gynecologist increased from around 73 days before the crisis to 109 days in 2014, and the proportion of patients waiting for more than a month rose from 36.7 to 41.5% [32, 33]. It has to be noticed that high waiting times were the main reason to declare unmet needs during the crisis, according to the Spanish National Health Surveys [34, 35]. Also, a recent study has been shown how pro-rich inequities in unmet needs increased in Spain along the economic crisis, as well as pro-rich inequities in access to screening tests such as mammography [36]. Some other studies have also identified an increase in pro-rich inequity related to publicly financed visits to specialists [37, 38]. All these facts suggest that social inequalities in cervical cancer screening could have increased over the Great Recession.

This paper explicitly estimates the degree of income-related inequality in cytology testing for Spanish women in 2006 and 2011 by employing concentration indices, and decomposes changes in inequality in order to ascertain how the contribution of each explanatory factor has evolved over time. This approach has important policy implications since it could improve the design of actions to prevent cervix cancer while coping with inequalities. An additional contribution of this paper resides in the analysis of a period marked by the impact of the economic crisis.

## Material and methods

### Material

Cross-sectional data from the Spanish National Health Survey (SNHS) were used, corresponding to two editions: years 2006–07 and 2011–2012. The SNHS is a representative survey of the Spanish population, carried out by the Spanish Statistical Office and coordinated by the Ministry of Health. The sample is stratified according to a three stages design: primary stage comprise the regions and subsequent stages census sections and main family dwellings. Data were collected throughout interviews conducted from June 2006 to June 2007 and from July 2011 to June 2012. The survey is structured in three different types of questionnaire: household, children and adults. Considering the purposes of this research, we chose data from the adults’ questionnaire filtering by women. We focused only on targeted women according medical guidelines about cervical cancer screening [12]. Therefore, we selected women from 25 to 64 years old. We also used socioeconomic data extracted from the household questionnaire.

### Methods

We used conventional methods for measuring and decomposing income-related inequality in cytology testing. In particular, in order to estimate inequality we employed the concentration index (CI) as proposed by Wagstaff et al. (1991) [39]. The CI is based on the concentration curve, which represents the relationship between the cumulative proportion of population ranked by income and the cumulative proportion of the variable of interest (in our case, use of cervical cytology during the period defined by medical guidelines of cervical cancer screening). The concentration index measures twice the area between the concentration curve and the 45° line. The CI can be calculated as follows:

1 $$ CI=\frac{2}{n^2\overline{y}}{\sum}_{i=1}^n{y}_i{r}_i $$

where $\overline{y}$ is the mean of the variable of interest, and r_i_ is the cumulative percentage that each individual represents over the total population once the latter has been ranked by income. The values of this index range from − 1 to 1, or from $\overline{y}-1$ to $1-\overline{y}$ when *y* is dichotomous [40]. A positive (negative) index would suggest a pro-rich (pro-poor) concentration of cytology testing and would be represented by a concentration curve below (above) the 45° line. If *CI* = 0, then there is no income-related inequality in the distribution of *y*, and the concentration curve coincides with the 45° line.

When there is a linear relationship between *y* and a set of k explanatory variables *x*: $y=\alpha +{\sum}_k{\beta}_k{\overline{x}}_k+e,$the CI may be expressed as a weighted sum of the partial concentration indices for the explanatory factors of inequality, being the weight the elasticity of *y* with respect to *x*_*k*_ [41]:

2 $$ CI=\sum \limits_k\left(\frac{\beta_k{\overline{x}}_k}{\overline{y}}\right){CI}_k+\frac{GCI_e}{\overline{y}} $$

Moreover, changes of CI over time (*∆CI* = *CI*_*t*_ − *CI*_*t* − 1_) may be disentangled by using an Oaxaca-type decomposition [41], such that variation of CI can be explained by changes in elasticities and by changes in *CI*_*k*_:

3 $$ \Delta  CI={\sum}_k\left(\frac{\beta_{k,t}{\overline{x}}_{k,t}}{{\overline{y}}_t}\right)\left({CI}_{k,t}-{CI}_{k,t-1}\right)+{\sum}_k{CI}_{k,t-1}\left(\frac{\beta_{k,t}{\overline{x}}_{k,t}}{{\overline{y}}_t}-\frac{\beta_{k,t-1}{\overline{x}}_{k,t-1}}{{\overline{y}}_{t-1}}\right)+\Delta  \frac{GCI_{et}}{\overline{y_t}} $$

Alternatively, since Oaxaca decomposition is not unique [41], the variation of CI may be expressed as:

4 $$ \Delta  CI={\sum}_k\left(\frac{\beta_{k,t-1}{\overline{x}}_{k,t-1}}{{\overline{y}}_{t-1}}\right)\left({CI}_{k,t}-{CI}_{k,t-1}\right)+{\sum}_k{CI}_{k,t}\left(\frac{\beta_{k,t}{\overline{x}}_{k,t}}{{\overline{y}}_t}-\frac{\beta_{k,t-1}{\overline{x}}_{k,t-1}}{{\overline{y}}_{t-1}}\right)+\Delta  \frac{GCI_{et}}{\overline{y_t}} $$

As non-linear models are adequate when the outcome variable is dichotomous, we used probit models to carry out all the estimates. Then, some linear approximation is needed to perform decomposition analysis. This can be done by substituting in eqs. (2)–(4) *β*_*k*_coefficients by $$ {\beta}_k^m $$, which are the partial effects ($$ \raisebox{1ex}{$ dy$}\!\left/ \!\raisebox{-1ex}{$d{x}_k$}\right.\Big) $$ evaluated at sample means.

Also, when the outcome variable is dichotomous, the concentration index has to be corrected in order to allow comparisons between groups of individuals from different time periods, that may show different levels of use of health services [42]. Erreygers suggests the following corrected concentration index: $E=\frac{4\overline{y}}{y^{max}-{y}^{min}} CI$, where *y*^*max*^and *y*^*min*^ are the bounds of *y*. When the Erreygers’ corrected index is used, the decomposition of inequality may be expressed by (5).

5 $$ E=4\cdot \sum \limits_k\left({\beta}_k^m{\overline{x}}_k\right){CI}_k+{GCI}_{\varepsilon } $$

which provides the same results as (2) [43], an also *∆E* provides the same results as (3)–(4).

### Definition of variables

Measurement of inequality through a concentration index and decomposition analysis requires a continuous variable that enables to rank individuals according to their socioeconomic status. However, the SNHS's display information about the net monthly income corresponding to each household as a categorical variable with eight and ten response intervals for 2006–07 and 2011–12 editions, respectively. Therefore, we had firstly to generate a continuous ranking variable from those categories. For the latest year we used the estimation of the household income by González-Almorox & Urbanos (2016) [44], calculated from the predictions of an interval regression model based on the information of the head of the household, where the covariates include gender, age, education, labor status, social class and region of residence. We replicated this method in order to compute household income for the year 2006. The modified OECD equivalence scale was used to calculate equivalent income. Following Siegel (2014) [45], equivalent income was rescaled to the price level of 2012 by using the consumer price indices provided by the Spanish Statistical Office.

Our variable of interest is based on the use of cervical cytology testing, which in the SNHSs was asked as follows: “Have you ever undergone a cervical cytology?” with a binary answer (yes/no). When the answer was ‘yes’, it was followed by the question “When did you undergo the latest one?”. Women reporting a previous cervical cytology were grouped according to the time since the latest cytology testing, based on the medical guidelines of cervical cancer screening: up to 3 years when they were aged between 25 and 34, and up to 5 years if they were 35 years old or over. Those women who didn’t comply with these requirements may be considered as underusers. Therefore, we defined our outcome variable as a dummy taking the value of 1 for the two former groups, and 0 for the latter.

According to the model proposed by Andersen (1995) [46], demand for health services depends on need, predisposing and enabling factors. Need is related to aspects of individuals’ health status. Predisposing factors include demographic characteristics and health beliefs, as well as educational level and labor status, whilst amongst enabling factors are those with influence on the access and use of health care, such as income level, place of residence and health insurance. The frontier between these two categories of factors is not always clear, since some of the so-called predisposing characteristics may also be considered as enabling. However, for our purposes it is not necessary a strict distinction between these groups. Our explanatory variables have therefore been selected following Andersen model, and are also based on previous literature, particularly on those studies focused on the analysis of social inequalities [47].

Socio-demographic characteristics were proxied by age, marital status and nationality. Physical inactivity during leisure time was employed to proxy health beliefs. Since no specific information about clinical status that could justify the need of cytology testing was available, we proxied health status by self-reported health, which has proved being a good predictor of morbidity and use of health services [48, 49]. As the preventive program is opportunistic, the visit to the doctor is a pre-requisite for the screening test being performed. Therefore, self-assessed health might be relevant to explain the probability of reporting a cervical cytology. We also included educational level, working status, (log of) equivalent household income, region of residence and a dummy variable indicating the type of her insurance scheme. We distinguished having only public coverage without direct access to specialist visits from having whether double (public and private) coverage or some of the public schemes financing direct access to private specialists.

Individual weights were applied to data to make the sample representative of the whole population. The statistical analysis was performed using StataSE 13 ©. The concentration indices were calculated by using the conindex command [50]. The final sample consisted of 10,743 observations in 2006–07 and 6587 in 2011–12. Table 1 shows the definition of the variables and descriptive statistics of the sample.

**Table 1:** Definition of variables and descriptive statistics of the sample

Variables	Definition	Mean 2006 (*n* = 10,743)	Mean 2011 (*n* = 6587)
Cytology	1 if woman underwent a cervical cytology in the latest 3 (if aged 25 to 34) or 5 (if aged 35 or over) years; 0 otherwise	73.9%	77.9%
25–34 (reference category)	1 if aged from 25 to 34; 0 otherwise	28.4%	25.6%
35–44	1 if aged from 35 to 44; 0 otherwise	29.5%	29.1%
45–54	1 if aged from 45 to 54; 0 otherwise	22.5%	25.6%
55–64	1 if aged from 55 to 64; 0 otherwise	19.6%	19.7%
Married	1 if married or living in couple; 0 otherwise	75.4%	64.3%
Spanish	1 if Spanish nationality; 0 otherwise	92.0%	85.6%
Ph_inact	1 if physically inactive in leisure time; 0 otherwise	40.3%	47.2%
Good_health (reference category)	1 if self-reported health is very good or good; 0 otherwise	66.6%	73.5%
Fair_health	1 if self-reported health is fair; 0 otherwise	25.6%	20.0%
Bad_health	1 if self-reported health is bad; 0 otherwise	5.7%	5.3%
Verybad_health	1 if self-reported health is very bad; 0 otherwise	2.1%	1.2%
Educ1 (reference category)	1 if woman has compulsory education or under; 0 otherwise	47.7%	45.4%
Educ2	1 if woman has non-compulsory secondary education or equivalent studies; 0 otherwise	21.9%	25.2%
Educ3	1 if woman has university or equivalent studies; 0 otherwise	30.4%	29.4%
Working	1 if woman is working; 0 otherwise	54.9%	57.5%
Ln_eqincome	Log of the equivalent household income (euros 2012)	6.86	6.67
Public_insurance	1 if woman only has public insurance without direct access to private specialists; 0 otherwise	79.7%	81.0%
Region_1	1 if resident in Andalusia; 0 otherwise	18.5%	17.5%
Region_2	1 if resident in Aragon; 0 otherwise	2.6%	2.8%
Region_3	1 if resident in Asturias; 0 otherwise	2.6%	2.3%
Region_4	1 if resident in Balearic Islands; 0 otherwise	3.0%	2.5%
Region_5	1 if resident in Canary Islands; 0 otherwise	5.2%	4.7%
Region_6	1 if resident in Cantabria; 0 otherwise	1.3%	1.3%
Region_7	1 if resident in Castilla-Leon; 0 otherwise	5.4%	5.3%
Region_8	1 if resident in Castilla-La Mancha; 0 otherwise	3.7%	4.0%
Region_9	1 if resident in Catalonia; 0 otherwise	12.6%	15.9%
Region_10	1 if resident in Valencia; 0 otherwise	11.1%	10.9%
Region_11	1 if resident in Extremadura; 0 otherwise	2.1%	2.1%
Region_12	1 if resident in Galicia; 0 otherwise	5.7%	5.9%
Region_13	1 if resident in Madrid; 0 otherwise	14.4%	14.6%
Region_14	1 if resident in Murcia; 0 otherwise	3.2%	3.1%
Region_15	1 if resident in Navarre; 0 otherwise	1.4%	1.4%
Region_16	1 if resident in Basque Country; 0 otherwise	6.2%	4.7%
Region_17	1 if resident in La Rioja; 0 otherwise	0.6%	0.7%
Region_18	1 if resident in Ceuta or Melilla; 0 otherwise	0.3%	0.2%

## Results

Table 1 shows the mean value of all the variables for both analyzed surveys. It is worth noting that the prevalence of women undergoing a cytology increased around 4 percentage points from 2006-07 (73.9%) to 2011–12 (77.9%). The comparison between both surveys also shows that the proportion of Spanish and married women decreased over time, as well as the household income level. Conversely, the proportion of women without private healthcare coverage, and the prevalence of physical inactivity and self-reported good health increased along the period. The rest of variables registered minor variations. However, it may be noticed that the proportion of population living in Catalonia and the Basque country show significant changes from one survey to another. In particular, according to the SHNS’s the percentage of Spaniards living in Catalonia would have significantly increased from 2006 to 07 to 2011–12, whilst the opposite would have happened in the Basque country.

According to our results, income-related inequality is statistically significant and favors the better-off in both periods. The corrected concentration indices point out that inequality significantly increased along the period, reaching 0.1726 in 2006–07 and 0.1880 in 2011–12 (*p* < 0.001).

Table 2 shows the contributions to inequality in cervical cancer screening for each year and the decomposition of total change. The three first columns for every year report the estimated partial effect retrieved from the probit model, the elasticity of cervical cancer screening for each explanatory variable and the concentration index for each regressor, respectively. Moreover, the fourth column shows the absolute contribution of each factor to overall income-related inequality, which is the product of the elasticity and the partial concentration index. A positive (negative) absolute contribution implies that, if inequality in cervical cancer screening was determined by that variable alone, then it would favor the better-off (worse-off). The fifth column reports the percentage contribution, which is obtained by dividing the absolute contribution by the overall income-related inequality (as measured by Erreygers CI). Finally, the last column of the table displays the absolute change in contributions from 2006-07 to 2011–12.

**Table 2:** Contributions to inequality in cervical cancer screening 2006–07 and 2011–12 and decomposition of total change

	2006–07					2011–12					Total change
Variables	Partial effect	Elasticity	Corrected CI	Contribution (1)	% Contribution	Partial effect	Elasticity	Corrected CI	Contribution (2)	% Contribution	Contribution (2)–(1)
35–44	0.1299***	0.0518	0.1097	0.0057	3.3%	0.0844***	0.0316	0.0285	0.0009	0.5%	−0.0048
45–54	0.1368***	0.0416	−0.1588	−0.0066	−3.8%	0.0857***	0.0281	−0.0157	− 0.0004	− 0.2%	0.0062
55–64	0.0383***	0.0102	− 0.3293	− 0.0033	−1.9%	− 0.0195	− 0.0049	0.1396	− 0.0007	− 0.4%	0.0027
Spanish	0.1463***	0.1819	0.0033	0.0006	0.3%	0.1354***	0.1489	0.0756	0.0113	6.0%	0.0107
Married	0.1603***	0.1635	−0.0568	− 0.0093	−5.4%	0.0644***	0.0532	−0.0346	− 0.0018	−1.0%	0.0075
Ph_inact	−0.0444***	− 0.0242	− 0.2233	0.0054	3.1%	−0.0630***	− 0.0381	− 0.2474	0.0094	5.0%	0.0040
Fair_health	0.0409***	0.0141	− 0.3630	−0.0051	−3.0%	0.0242	0.0062	−0.3064	− 0.0019	−1.0%	0.0032
Bad_health	0.0293	0.0022	−0.5187	− 0.0012	− 0.7%	− 0.0345	− 0.0023	−0.5829	0.0014	0.7%	0.0025
Verybad_health	0.0531	0.0015	−0.3333	−0.0005	− 0.3%	0.0633	0.0010	−0.7164	− 0.0007	− 0.4%	− 0.0002
Working	0.0316***	0.0235	0.4497	0.0106	6.1%	0.0395***	0.0292	0.4778	0.0139	7.4%	0.0034
Educ2	0.0404***	0.0120	0.2691	0.0032	1.9%	0.0478***	0.0155	0.1315	0.0020	1.1%	− 0.0012
Educ3	0.0463***	0.0190	1.1101	0.0211	12.2%	0.0801***	0.0302	1.0825	0.0327	17.4%	0.0116
Ln_eqincome	0.1110***	1.0295	0.1053	0.1084	62.8%	0.0711***	0.6094	0.1336	0.0814	43.3%	−0.0270
Public_ins	− 0.1122***	− 0.1209	− 0.2616	0.0316	18.3%	− 0.0471**	− 0.0490	− 0.2434	0.0119	6.3%	− 0.0197
Region2	0.0610***	0.0021	0.3763	0.0008	0.5%	0.0241	0.0009	0.7786	0.0007	0.4%	−0.0001
Region3	0.0408	0.0014	0.2910	0.0004	0.2%	0.0077	0.0002	0.3192	0.0001	0.0%	−0.0003
Region4	0.1124***	0.0045	0.7439	0.0034	1.9%	0.0905***	0.0029	0.3848	0.0011	0.6%	−0.0023
Region5	0.1155***	0.0081	−0.7660	−0.0062	−3.6%	0.1613***	0.0096	−0.9279	−0.0090	−4.8%	− 0.0027
Region6	−0.0031	− 0.0001	−0.2263	0.0000	0.0%	0.0000	0.0000	−0.0415	0.0000	0.0%	0.0000
Region7	0.0582**	0.0043	−0.2247	−0.0010	− 0.6%	0.0947***	0.0065	0.2429	0.0016	0.8%	0.0025
Region8	0.0071	0.0004	−0.4940	− 0.0002	−0.1%	0.0600**	0.0031	−0.6753	−0.0021	−1.1%	− 0.0019
Region9	0.0285	0.0049	0.8364	0.0041	2.4%	0.1326***	0.0270	0.6263	0.0169	9.0%	0.0129
Region10	0.0089	0.0013	0.0693	0.0001	0.1%	0.1101***	0.0155	−0.1839	−0.0028	−1.5%	−0.0029
Region11	−0.0369	−0.0011	−1.0699	0.0011	0.7%	0.0196	0.0005	−0.6949	−0.0004	− 0.2%	−0.0015
Region12	0.0400**	0.0031	−0.3635	−0.0011	− 0.7%	0.0213	0.0016	−0.4345	− 0.0007	−0.4%	0.0004
Region13	0.0425**	0.0083	0.6799	0.0056	3.3%	0.0955***	0.0180	0.4617	0.0083	4.4%	0.0027
Region14	0.0308	0.0013	−0.5062	−0.0007	− 0.4%	0.0824***	0.0033	−0.4122	− 0.0013	−0.7%	− 0.0007
Region15	0.0963***	0.0018	0.6742	0.0012	0.7%	0.0300	0.0005	1.0422	0.0005	0.3%	−0.0007
Region16	0.0786***	0.0066	0.4933	0.0033	1.9%	0.0605**	0.0037	0.8995	0.0033	1.8%	0.0001
Region17	0.0095	0.0001	0.1690	0.0000	0.0%	0.0976***	0.0008	0.4331	0.0004	0.2%	0.0003
Region18	−0.0554	−0.0002	−0.5003	0.0001	0.1%	0.0432	0.0001	−0.8504	−0.0001	0.0%	−0.0002
Residual				0.0011	0.6%				0.0122	6.5%	0.0111
Corrected CI				0.1726	100.0%				0.1880	100.0%	0.0154

*CI*: concentration index

***Statistically significant at 99% level (*p* < 0.01); **Statistically significant at 95% level (*p* < 0.05)

The partial effects shown in Table 2 indicate that, for both surveys, 35–54 years old, Spanish, working and married or living in couple women showed a significantly higher probability of cervical cancer screening, in comparison to those younger, foreigner, not working or living without a couple. Also, income and educational level were highly and positively associated with the use of cervical cancer screening. Conversely, being physically inactive in leisure time and lacking direct access to private specialists reduced the probability of cytology testing. Self-assessed health only appeared to be significant in 2006–07, when reporting fair health was positively related to the use of preventive services, compared to reporting good or very good health. There are also some other significant changes in the partial effects across the period. In particular, age and marital status showed a lower influence on cervical screening in 2011–12 compared to 2006–07, as well as the household income. However, having university studies was much more influent at the end of the period. Futhermore, most of regional dummies showed different effects from one period to another: in eight of them it was observed a noticeable increase in the partial effect, whilst a remarkable decrease was registered in other five.

Negative (positive) signs for corrected concentration indices in Table 2 indicate that the explanatory variables had a pro-poor (pro-rich) distribution. Most variables showed the expected sign. It is worth noting the change registered in the sign for the aged 55–64: the previously pro-poor distribution became pro-rich with the crisis, since this age group was relatively well covered by public benefits (mainly unemployment benefits that after expiring turned into retirement pensions), compared to the rest of population. Also, the significant reduction in the pro-rich distribution of women aged 35–44 reflects the strong impact of the economic recession on young cohorts. Some other variables registered observable changes in their concentration indices across the analyzed period, such as non-compulsory education (showing a decrease in its pro-rich distribution), nationality and income (both showing an increase). Further, according to the concentration index of the dummy *verybad_health*, income-related inequalities in self-reported health seem to have suffered a remarkable rise across time.

According to our results, inequality in cytology testing was mainly explained by the direct effect of income in both periods, which accounted for 62.8% of the total in 2006–07 and for 43.3% in 2011–12. The (highly) negative contribution of income to total change shown in the last column of Table 2 indicates that its share in total inequality had (notably) decreased, despite the rise of inequality during the crisis. This is due to the lower elasticity of cervical screening with respect to income at the end of the period. The same effect is observed for having public insurance without direct access to private specialists. The type of health insurance, the educational level and the place of residence were ranked as the second (18.3%), third (14.1%) and fourth (6.3%) determinants of inequality, respectively, before the crisis started. However, in 2011–12 the rank of determinants slightly varied; the educational level occupied the second place (18.5%), followed by the region of residence (8.7%) and the working status (7.4%). All these factors tended to favor the better-off in both periods. This is also the case of nationality, whose contribution registered the highest increase (from 0.3 to 6%). The unexplained part of inequality, although higher in the final year compared to the beginning of the period, was low in both analyzed years, what indicates a good specification of the probit models.

Finally, Table 3 allows disentangling if changes over time were due to changes in elasticities of cytology testing with respect to its determinants, or due to changes in the concentration indices of regressors. For the age dummy 35–44, the type of insurance and some regions (the Balearic and the Canary Islands, and also Castilla-La Mancha), the changes in both components acted in the same direction, and tended to reduce pro-rich inequalities. For the age dummy, the main responsible of that effect was the significant decrease in the concentration index, whilst for the rest of the above-mentioned variables, the variation in their contribution to total inequality was due to changes in elasticities.

**Table 3:** Oaxaca-type decomposition for change in inequality (2006–2012)

	Equation (3)		Equation (4)		Total	
	*∆*CI *Elasticity	*∆*Elasticity*CI	*∆*CI *Elasticity	*∆*Elasticity*CI	Total	%
35–44	−0.0027	−0.0020	−0.0043	−0.0005	− 0.0048	−31%
45–54	0.0043	0.0019	0.0060	0.0002	0.0062	40%
55–64	−0.0024	0.0051	0.0047	−0.0020	0.0027	17%
Spanish	0.0107	−0.0001	0.0125	−0.0018	0.0107	69%
Married	0.0013	0.0061	0.0039	0.0035	0.0075	48%
Ph_inact	0.0005	0.0036	0.0003	0.0038	0.0040	26%
Fair_health	0.0005	0.0028	0.0010	0.0022	0.0032	21%
Bad_health	0.0001	0.0024	−00001	0.0026	0.0025	16%
Verybad_health	−0.0004	0.0002	−0.0005	0.0003	−0.0002	−1%
Working	0.0001	0.0032	0.0001	0.0033	0.0034	22%
Educ2	−0.0024	0.0012	−0.0017	0.0005	−0.0012	−8%
Educ3	−0.0026	0.0142	−0.0016	0.0131	0.0116	75%
Ln_eqincome	0.0138	−0.0408	0.0222	−0.0492	−0.0270	− 176%
Public_ins	−0.0016	− 0.0181	− 0.0037	−0.0160	− 0.0197	− 128%
Region2	0.0003	−0.0005	0.0008	−0.0009	− 0.0001	−1%
Region3	0.0000	−0.0004	0.0000	−0.0004	−0.0003	−2%
Region4	−0.0011	−0.0011	− 0.0017	−0.0006	− 0.0023	−15%
Region5	−0.0012	− 0.0016	−0.0009	− 0.0018	−0.0027	−18%
Region6	0.0000	0.0000	0.0000	0.0000	0.0000	0%
Region7	0.0031	−0.0006	0.0020	0.0006	0.0025	16%
Region8	−0,0005	−0.0014	− 0.0001	−0.0019	− 0.0019	−12%
Region9	−0.0069	0.0197	−0.0012	0.0140	0.0129	84%
Region10	−0.0040	0.0010	−0.0003	−0.0026	− 0.0029	−19%
Region11	0.0002	−0.0017	−0.0004	− 0.0011	−0.0015	−10%
Region12	−0.0001	0.0005	−0.0002	0.0006	0.0004	3%
Region13	−0.0046	0.0073	−0.0020	0.0047	0.0027	17%
Region14	0.0004	−0.0011	0.0002	−0.0008	−0.0007	−4%
Region15	0.0002	−0.0008	0.0006	−0.0012	−0.0007	−4%
Region16	0.0014	−0.0013	0.0024	−0.0023	0.0001	0%
Region17	0.0002	0.0001	0.0000	0.0003	0.0003	2%
Region18	0.0000	−0.0002	0.0001	−0.0003	− 0.0002	−1%
Residual					0,0111	72%
Total	0.0068	−0.0025	0.0379	−0.0336	0.0154	100%

*CI*: concentration index

A second group of variables were those for which changes in elasticities and in concentration indices also acted in the same direction, but tended to increase pro-rich inequalities: this was the case for those variables representing women between 45 and 54 years old, marital status, physical inactivity, fair and bad health, and working status. Again, the registered changes in their contribution to inequality were mostly due to the impact of elasticities, except for the age variable.

Lastly, changes in elasticities and in concentration indices acted in different directions for those variables representing women between 55 and 64 years old, nationality, very bad health, educational level, income and most Spanish regions. The final effect implied that very bad health and income contributed to reduce pro-rich inequalities over time, whilst the rest of determinants of cervical screening contributed, in global terms, to their increase. The leading effect was due to changes in elasticities for the age dummy, educational level and income. The same holds true for Catalonia, the region showing the highest contribution to the increase of inequality over time. Conversely, the final impact of nationality and very bad health was mainly due to changes in their concentration indices.

According to the percentages shown in the last column of Table 3, income and the type of health insurance notably contributed to a reduction of pro-rich inequalities in cervical screening. However, their effect was more than compensated by the role played by other factors, such as nationality and the educational level. Also, the absolute contribution of the error term to the overall change of inequality significantly increased over time, since it accounted for 72% of total change, what implies that a meaningful part of the variation of inequality still remains unexplained.

## Discussion

Our results show that income-related inequalities in cervical screening, which favor the better-off, significantly grew up from 2006-07 to 2011–12. This goes in line with some previous studies showing that Spanish pro-rich inequities in access to specialist doctors were intensified over the economic crisis [37, 38], and also pro-rich inequalities in some other screening tests [36]. However, our results indicate as well that the prevalence of cytology testing in Spain increased during the period 2006–2012, which is consistent with previous findings [30]. The descriptive statistics of our sample also show changes in other variables that deserve some comment. For instance, the proportion of Spanish women notably decreased over time. Although the migration flows began to reverse after the economic crisis came up, the composition of population in 2011–12 significantly had changed compared to the previous decade, and the proportion of non-Spaniards had accordingly increased [51]. The crisis impact also may be seen in the reduction of household income level and the slight increase of the proportion of women without private health coverage. Surprisingly, the prevalence of working women slightly grew up. This is compatible with a high increase of the unemployment rate for our sample of women (from 9.9% in 2006–07 to 16.7% in 2011–12), since what happened was that the category of students and homemakers notably reduced during the analyzed period (from around 28 to 21%). Also, the proportion of women reporting good or very good health significantly increased over time. It has been suggested that this fact could be due to that, during the Spanish crisis, other priorities ranked first compared to health [52]. Lastly, it can be checked that the regional distribution of population in the SNHS 2006–07 is slightly different from official population statistics for that period. This problem only affects to Catalonia (that appears with a percentage of population lower than expected) and the Basque country (with a percentage higher than expected). This fact could be due to differences in the sample design for population statistics and health surveys.

The estimations obtained from our probit models are highly consistent with previous literature, which associates cervical screening with higher social status [22–28, 30], educational level [14, 22, 24, 25, 30, 53–57], self-perception of bad health [58], middle-old age and not being foreign [25, 26, 28, 30, 53–55, 58, 59], having a partner or being married [14, 24, 25, 28, 30], being employed [14, 24] or having private health insurance [24, 25, 27, 28, 53, 55, 60, 61]. We also found that pro-rich-inequality is mainly explained by socioeconomic factors in both analyzed years, with income, educational level and working status playing an essential role in total inequality, in line with previous evidence about the patterns of use of preventive health care services [13, 27].

After measuring income-related inequality in cervical screening and calculating the contribution of each relevant factor for both years, we used an Oaxaca-type decomposition in order to distinguish the effect of changes in elasticity of cytology testing with respect to its determinants, on one hand, from the impact of changes in inequalities in these factors, on the other. According to our results, change in inequalities is mainly due to changes in elasticities. That means that, despite the crisis, the distribution along income of most of the variables used in the analysis hardly varied from the beginning to the end of the period, although the influence of some factors significantly did. In particular, we found a stronger influence of higher education on the use of cervical screening and a decrease in the contribution of income. This fact could be pointing to the relevance of access to and ability to process information over other economic factors, such as the ability to pay, in the context of a National Health Service.

Also, the crisis seems to have significantly increased the influence of nationality on income-related inequality in cervical screening. At this point, it should be mentioned that the Spanish Government revoked previous full right to public health care coverage for undocumented migrants and some other groups through the Royal Decree-Law 16/2012, although it was unequally implemented by regional governments [62]. However, despite the Decree entered into force before the data collection of SNHS 2011–12 had finished, this reform is expected to have a low impact on our results.

Nevertheless, we found that the influence of having direct access to private specialists noticeably decreased from 2006-07 to 2011–12, despite the rise in public waiting lists. This could be interpreted as a loss of relevance of some factors representing direct access barriers to health care, such as waiting time, in opposition to the role played by more subtle barriers, such as education or nationality.

Finally, we have shown that the region of residence has a not negligible influence on income-related inequality in cervical screening. Nevertheless, our results don’t seem to be systematically related to regional income, political sign or any other relevant variable at a regional level. Therefore, disentangling regional effects would deserve further research. Differences in contributions to inequality by regions might be related to the different response that every region gave to the crisis in terms of spending cuts and implementation of reforms promoted by the central government, which would be added to the previous significant differences in health care budget and management.

Our study has a number of limitations. Firstly, our estimated income variable may introduce some bias in the analysis, although we cannot predict in which direction inequality may be affected by the potential biases. Also, we restricted the analysis to a period when the crisis showed its biggest impact. More recent data are available for years 2014 and 2017, provided by the European Health Interview Survey (EHIS) and the new edition of the SNHS, respectively. However, both years correspond to a post-crisis period. Additionally, the information provided by the EHIS, which is referred to the first year of economic recovery, is not completely comparable to that retrieved from the SNHS.

Additionally, changes in elasticities shown in Table 2 could be further decomposed by using the total differential approach proposed by Wagstaff et al. (2003) [41], in order to disentangle the effect of changes in the coefficients and the means of the regressors. We also performed this analysis, but since high approximation errors were obtained for most variables, we finally dismissed the results. It should be reminded that the total differential approach is only accurate for small changes, as it is based on an approximation. Moreover, it should be noted that, after the Oaxaca-type decomposition, an important part of the change in inequality remains unexplained. It could be suggested that some structural factors and contextual trends which are not captured by survey data could be explaining changes over time [63], particularly if we consider that some major economic and social changes took place during the analyzed period. Also, other potential relevant variables such as wealth, which is not available in our dataset, could provide some explanatory power. However, and despite these limitations, our analysis still provides valuable insight about the factors behind the evolution of income-related inequality in cytology testing during the hardest years of crisis in Spain.

## Conclusions

Since the economic crisis seems to have intensified the situation of vulnerability of migrants and other population groups, such as low educated, the reduction of cervical screening inequalities would require focusing on these specific groups. The reestablishment of full rights to public healthcare coverage, already approved by the new Spanish Socialist Government [64], could contribute to this goal. Also, the influence of socioeconomic variables (including income) may be counterbalanced by the implementation of population-based screening programs. Until now, very few papers estimate the expected cost-benefit/cost-effectiveness ratio of these kind of programs, which is crucial to provide useful guidance about how they should be designed [65, 66]. However, it would be needed to pay more attention to this issue in order to develop screening programs which simultaneously fulfill efficiency and equity criteria.

## Abbreviations

CCI: Corrected concentration indices
CI: Concentration index
EHIS: European Health Interview Survey
SNHS: Spanish National Health Survey
